# Advancing Cardiovascular Disease Prevention, Management, and Control Through Field Epidemiology Training Programs in Noncommunicable Diseases in Low- and Middle-Income Countries

**DOI:** 10.5888/pcd20.220215

**Published:** 2023-04-27

**Authors:** Sushama D. Acharya, Qaiser Mukhtar, Patricia Richter

**Affiliations:** 1 Office of Global Noncommunicable Diseases, Division of Global Health Protection, Centers for Disease Control and Prevention, Atlanta, Georgia; 2 Graham Technologies, LLC, Upper Marlboro, Maryland

## Introduction

Since 1980, the Centers for Disease Control and Prevention (CDC) has worked closely with partners worldwide to protect population health through Field Epidemiology Training Programs (FETPs). These programs are country-owned, on-the-job training programs that collaborate with local mentors and partners to focus on evidence-based best practices and research methods. The FETP goal is to build the global workforce of field epidemiologists, or “disease detectives,” and to increase their ability to detect and respond to health threats, address the severe worldwide shortage of skilled epidemiologists, and build critical relationships among partnering countries (1). The program offers 3 tiers of training, and each country can select the tier best suited to their needs: 1) FETP Frontline, which works at the local and community level; 2) FETP Intermediate, which has a regional focus; and 3) FETP Advanced, which prepares health professionals for leadership roles in ministries of health and other national-level government agencies. In all 3 tiers, 25% of training is condensed classroom instruction, and 75% is hands-on training in the field to gain experience and competence in field epidemiology (1). 

Until recently, FETP training focused on infectious disease response, despite the growing burden of noncommunicable diseases (NCDs), which are responsible for 74% of all deaths worldwide (2). Of all NCD deaths, 77% are in low- and middle-income countries (LMICs) and 86% of premature deaths among people aged 30 to 69 years in LMICs are attributable to NCDs (2).

The COVID-19 pandemic has amplified the global need for an NCD-trained public health workforce. People with NCDs have a higher risk of becoming severely ill with COVID-19 and are more likely to die from it (3). Therefore, building applied technical capacity in NCD epidemiology and surveillance is critical to inform decision makers about the health risk posed by COVID-19 among people with NCDs. However, 1% or less of all global health funding is invested in NCD prevention (4). For example, deaths from 1 NCD alone, cardiovascular disease (CVD), exceed all communicable disease deaths combined. Addressing NCDs with a focus on CVDs and their risk factors in LMICs supports stronger global economies, health security, and progress toward the United Nations’ Sustainable Development Goals of reducing NCD premature mortality by one-third by 2030 (5).

## Field Epidemiology Training Program Noncommunicable Disease Tracks

In 2018, CDC and global partners established 2-year advanced level FETP NCD training tracks in China, Ethiopia, India, and Thailand to create a workforce to address leading NCDs with a focus on CVDs. Worldwide, CVDs account for 18.5 million annual deaths, but when detected early, their risk factors can be managed and treated (6). Although NCD-track trainees learn and apply skills in CVD epidemiology and associated risk factors, these same skills apply to other NCDs and their risk factors.

While CDC serves as the technical lead, the FETP NCD tracks are supported by the Bloomberg Philanthropies and Resolve to Save Lives (RTSL) through a grant to the National Foundation for the Centers for Disease Control and Prevention (CDC Foundation). The CDC Foundation provides support in administering the grant for FETP NCD program activities. The Training Programs in Epidemiology and Public Health Interventions Network (TEPHINET), a professional organization with a reach of more than 100 countries, is also a key partner in recognizing the need to build FETP NCD capacity to address the growing burden of NCDs. TEPHINET promotes the program, provides the platform for program activities, and disseminates resources to FETPs. Local partners include ministries of health, FETPs, universities, and local CVD experts, such as in-country RTSL health facilities and affiliates.

Four CDC technical and programmatic principles support FETP NCD tracks in the 4 participating countries. We highlight selected accomplishments and available technical resources developed to strengthen NCD capacity in the relatively new FETP NCD program. We offer recommendations for creating FETP NCD programs in additional countries and establishing sustainable programs.

## Four Principles and Key Accomplishments of the FETP NCD Track

Four principles support global FETP NCD capacity building: 1) program implementation, 2) curricula development and training, 3) continuous professional development, and 4) building research and publication capacity. The eligibility criteria and graduation requirements for trainees are specific to each of the 4 countries currently participating (Table 1). Trainees across all 4 countries spend most of their field time “learning by doing,” completing field projects with technical guidance from local and global mentors. Required field projects generally include surveillance data analysis, field and outbreak investigations, evaluation of surveillance systems and/or programs, planning and conducting epidemiologic studies, and disseminating findings.

**Table 1:** Noncommunicable Disease (NCD) Field Epidemiology Training Program (FETP), Advanced-Level Program, Structure and Accomplishments

Program structure and accomplishments	Country			
	China	Ethiopia	India	Thailand^a^
FETP NCD track launch, y	2019	2019	2018	2018
Program description	2-year training within China CDC	2-year training within Ethiopia Public Health Institute	2-year training within ICMR	2018–2020: 2-year training within the Ministry of Public Health
Qualification of FETP NCD trainees	Completed medical training or a graduate degree (eg, MPH, PhD) and some NCD experience	Students working toward MPH with interest in NCD epidemiology	Physicians from the Ministry of Health working on NCDs	Physicians with interest in NCD epidemiology
FETP NCD mentorship	3 Mentors: primary, program, and field	3 Mentors: academic, field, and RTSL program coordinator	2 Mentors: primary and secondary	2 Mentors: primary and secondary/ project-based
Local FETP NCD partners	China RTSL	Ethiopia RTSL	India RTSL	Thailand WHO
Technical exchange attended	2 in-person and 2 virtual	2 in-person and 2 virtual	3 in-person and 2 virtual	3 in-person and 2 virtual
No. of FETP NCD field projects completed	49	54	32	13
No. of FETP NCD trainings	22	9	10	4
No. of FETP NCD graduates	15	7	2	7

Abbreviations: CDC, Centers for Disease Control and Prevention; ICMR, Indian Council of Medical Research; RTSL, Resolve to Save Lives; WHO, World Health Organization.

a In 2020, Thailand switched to an integrated model, giving all FETP trainees the option to conduct 1 or more NCD field projects.

1. **Program implementation.** Program implementation support to the 4 funded countries consists of ongoing guidance, supportive tools, and technical resources aligned with FETP graduation requirements (Table 1). Countries participating in an FETP are encouraged to establish partnerships with local NCD organizations to strengthen mentorship and create sustainability. Program participants engage in annual technical exchanges with other countries implementing FETPs to foster cross-collaboration and peer learning opportunities, and they monitor challenges, explore solutions, document best practices, and disseminate success stories to highlight the contributions of FETP NCD trainees (7). FETP trainees receive standardized competency-based CVD-specific training, workshops, and subject matter expertise to help them complete graduation requirements. Although NCD tracks are supported in 4 countries, all technical tools and professional development opportunities are open to FETPs in any country at no cost.
2. **Curricula development and training**. Modern curricula and tools aligned with core FETP standards are available to teach skills in collecting, analyzing, and disseminating data for CVD prevention, management, and control. Although training focuses on CVDs, learned skills can be applied to other NCDs. In addition, to make information resources more accessible, we are replacing didactic in-person training with online self-study training materials, which will be available at no cost to trainees and mentors. Online materials have mobile responsiveness features to mitigate technological barriers. They also can be adapted to a facilitator-led, in-person, or hybrid teaching approach, combining online self-study courses with facilitator-led hands-on exercises and case studies. Additionally, the updated curricula listed below incorporate adult-learning principles to encourage interactive engagement, enhanced understanding, and knowledge retention:
  - **Online NCD epidemiology, surveillance, and data analysis.** Four online NCD epidemiology courses are available and 2 more are in development (Table 2).

**Table 2:** Curricula Focused on Noncommunicable Diseases (NCDs) Available to Field Epidemiology Training Programs (FETPs)

Curricula category	Access	Title
NCD epidemiology	Available on request	1. NCDs: Descriptive Epidemiology & Data Analysis 2. NCDs: Evaluating Surveillance System 3. NCDs: Evaluating Public Health Programs 4. NCDs: Writing an Epidemiological Research Protocol
Scientific communication	Online	1. Writing a Scientific Manuscript (available in English and Spanish) 2. Conducting a Literature Search 3. Developing a Poster Presentation 4. Developing and Delivering Effective Oral Presentations (coming soon)
Hypertension-focused case studies	Available on request	1. Effectiveness of a Clinic-Based Intervention Designed to Reduce Undiagnosed Hypertension in Country X 2. Missed Opportunities for Hypertension Control in Country X 3. Hypertension Screening and Treatment in Country X: Getting Started 4. Evaluation of Hypertension Screening and Treatment Efforts in District X: Information for Improvement 5. Cohort Study of Cardiovascular Disease Risk Factors in a Rural Area of Country X
Examining comorbidities during COVID-19 investigation (case studies)	Online (available in Arabic, English, French, Portuguese, and Spanish)	Field Epidemiology Training Program: Noncommunicable Disease COVID-19 Toolkit^a^ 1. Intermediate FETP Case Study: Investigating a Post-Pandemic Ischemic Stroke Surge at Capital City Hospital – Collecting, Reviewing, Interpreting, and Summarizing Data on Stroke and Associated Cardiovascular Disease Risk Factors Part A – Investigating Stroke Cases Part B – Investigating Risk Factors 2. Advanced FETP Case Study: An Epidemiological Study to Examine Stroke Hospitalizations During the COVID-19 Pandemic – Planning and Conducting Analysis Part A – Study Design Part B – Performing Analyses
Leadership and management	Available on request (prerecorded lectures)	1. Stakeholder Engagement (27 min) 2. Making a Case for Your Project (23 min) 3. Program Planning (part 1: 24 min; part 2: 27 min) 4. Conflict Resolution (30 min) 5. Effective Supervision and Performance Management (part 1: 18 min; part 2: 20 min) 6. Self-management (23 min) 7. Program Management during Emergencies (27 min) 8. Leadership Style (35 min)

Abbreviations: NCD, noncommunicable disease.

^a^ The toolkit also includes 1) field project topics, questions, areas for investigation, and suggested examples and 2) a literature synthesis report.
  - **Online scientific communication.** In 2020, an online course on writing a scientific manuscript was created and posted on the TEPHINET website (https://www.tephinet.org/scientific-writing-training-multiple-languages). This course is also available in Spanish and has registered more than 41,000 visits from more than 170 countries. Additionally, in March 2023, two online self-study courses — conducting a literature search and developing a poster presentation — were added to the scientific communication series (https://www.tephinet.org/tephinet-learning-center/tephinet-library). The course on developing and delivering effective oral presentations is forthcoming on the same website (Table 2). These interactive courses help FETP trainees learn and apply principles of effective communication to highlight critical findings for informing diverse audiences.
  - **NCD case studies**: Seven case studies are available as interactive teaching tools to show the application of epidemiologic theory or concepts to NCD-specific situations. Five case studies are hypertension-related, and 2 integrate assessment of NCDs during a COVID-19 investigation, offering all FETP trainees an opportunity to develop practical and critically needed NCD skills (Table 2). Participating FETPs interested in hypertension-related case studies can submit a request electronically (globalncds@cdc.gov); 2 case studies on the integration of NCDs and infectious diseases are posted on the TEPHINET website in 5 languages (Arabic, English, French, Portuguese, and Spanish) (https://www.tephinet.org/tephinet-learning-center/tephinet-library/field-epidemiology-training-program-fetp-noncommunicable).
  - **Leadership and management.** In response to requests from FETPs, a series of prerecorded lectures on various aspects of leadership and management have been developed (Table 2). Participating FETPs interested in these recorded lectures can request them electronically (globalncds@cdc.gov).
3. **Continuous professional development**. Ongoing learning opportunities for FETP mentors, trainees, and graduates include webinars, workshops, conference sessions, and a community of practice. For example, since November 2020, twenty-nine webinars have been offered on NCD-related topics, and more than 6,700 attendees from more than 115 countries participated in them. Last year, a synchronous, 8-part virtual webinar series on manuscript writing proved successful, averaging more than 500 participants per session. More than 160 participants attended 7 or more of these sessions, confirming the vast reach and effectiveness of the virtual teaching delivery mode. In addition, partnering programs are invited to join an online NCD community of practice called NCD Collaborative hosted on TEPHIConnect, which further promotes local and international networking.
4. **Building research and publication capacity.** Although the FETP NCD tracks are established in 4 countries, 2 programs allow all FETPs to compete for CVD research and publication mentorship opportunities:
  - **FETP Cardiovascular Disease Small Grants Program.** These grants aim to build FETP research capacity to promote locally generated evidence. Selected trainees receive up to $5,000 to conduct projects on clinical and community efforts to address CVD and associated risk factors. Selected applicants are matched with a remote CDC mentor for conducting CVD projects. Additionally, awardees receive support from an in-country mentor who works collaboratively with global mentors. Since 2018, this program has supported 36 projects from 14 countries.
  - **The Emerging Authors Program for Global Cardiovascular Disease Research (EAP):** EAP provides publication mentorship for early- and mid-career public health trainees and practitioners from LMICs. Selected authors receive guidance and support from remote global mentors to strengthen their scientific writing skills and navigate the writing and publication process. Mentors guide authors in defining study components, describing methods, presenting study results, discussing public health implications, and publishing. EAP is an excellent example of advancing the global health equity agenda, particularly in reducing disparities in global health research and evidence generation. Since 2019, EAP has led to 24 publications from 8 countries and 2 regions.

## Implications for Public Health Practice

The COVID-19 pandemic has revealed the devastating inequities in NCD funding and workforce training to address NCDs. Although NCDs are an emerging global health threat, there is a disconnect between policies and funding allocation for them compared with infectious diseases and epidemic risk (8). Addressing NCDs’ devastating effects on population health requires field epidemiologists trained in NCD-focused epidemiology, surveillance, data collection, and data synthesis to provide decision makers with timely evidence. However, establishing a specialized NCD track is resource-intensive and requires time and commitment. Although access to the developed NCD curricula offers interested FETPs technical resources, this should be accompanied by an understanding of in-country assets and barriers to sustaining NCD track implementation. Key challenges include identifying and retaining local NCD experts to mentor trainees and recent graduates. Competing priorities and field deployments can lead to trainees discontinuing the program. In addition, the absence of dedicated funding, strong support for NCDs, and NCD career paths deter FETP trainees from joining or completing the NCD track.

Since 2018, fifteen advanced-level NCD track cohorts enrolled 55 trainees across 4 countries; 31 have graduated, and 5 discontinued the program for personal or professional reasons. In addition, NCD trainees conducted more than 145 field projects, generating evidence on CVDs and their risk factors. Despite challenges, the presence of NCD programs has increased awareness, collaboration with NCD agencies, and a desire to expand NCD capacity-building. In response to the growing demand for NCD advanced tracks, we encourage interested countries to assess country-specific commitment and the resources needed to support NCD tracks to determine the best way forward. For example, countries with a strong political and financial commitment to addressing NCDs and cohesive NCD partnerships could successfully implement an advanced NCD track. On the other hand, FETPs with limited NCD funds but strong NCD partnerships and skilled mentors could offer FETP trainees the option to conduct NCD field projects without creating a specialized NCD track. At a minimum, FETPs can integrate NCD skill-building into their core training by using available NCD resources (Table 2). Finally, participating countries with established NCD tracks can scale their efforts through a train-the-trainer approach to expand NCD skill-building opportunities to state, province, or district levels. This approach could build a local pool of NCD trainers to expand capacity and reach. Countries can also consider integrating NCD tracks in intermediate training programs.

### Conclusion

Building FETP NCD capacity is essential to public health. We have highlighted CDC’s technical support for NCD tracks and potential challenges countries may face in implementing the tracks while offering realistic solutions and approaches to consider. The timely dissemination of this essay can raise awareness and introduce interested FETPs to available cost-free NCD training materials while opening doors for expanded collaboration and innovative implementation opportunities.
